# Change in macular thickness after uncomplicated phacoemulsification surgery using optical coherence tomography in a tertiary care hospital

**DOI:** 10.12669/pjms.39.5.4775

**Published:** 2023

**Authors:** Mehboob Dad, Muhammad Ali Tahir, Alyscia Cheema, Hina Nasreen Nawaz

**Affiliations:** 1 Mehboob Dad, MBBS. Fourth Year Resident, Training in Ophthalmology Department, Jinnah Post Graduate Medical Centre, Karachi, Pakistan; 2 Muhammad Ali Tahir, MBBS, FCPS (Ophthalmology), FCPS (Vitreoretina). Consultant Retinal Surgeon, Jinnah Post Graduate Medical Centre, Karachi, Pakistan; 3 Alyscia Cheema, MBBS, FCPS, FRCS. Professor of Ophthalmology, Jinnah Post Graduate Medical Centre, Karachi, Pakistan; 4 Hina Nasreen Nawaz, MBBS, MRCS. Jinnah Post Graduate Medical Centre, Karachi, Pakistan

**Keywords:** Macular thickness, Swept source optical coherence tomography, phacoemulsification

## Abstract

**Objectives:**

To determine the change in macular thickness after uncomplicated phacoemulsification surgery using optical coherence tomography in a tertiary care hospital.

**Methods:**

This study was conducted at Department of Ophthalmology Jinnah Postgraduate Medical Centre Karachi, for 6 months from 10^th^ Dec 2019 to 10^th^ June 2020. Sample size calculation of 52 eyes was done by using open epi software. Patients who fulfilled the inclusion criteria that is age ranging from 50 to 75 years, either gender with senile cataract having no preexisting ocular or systemic disease and those giving consent were included in this study. Patients with any comorbidity ocular trauma, having pre-existing ocular diseases such as active ocular infection, glaucoma, maculopathy or retinopathy were excluded from study. Patients with secondary cataract also excluded from study. After complete history, all patients underwent detailed ophthalmologic examination and Pre-surgery macular thickness recorded by using swept source OCT (DRI-OCT-2 Triton; Topcon). Surgery was performed and intraocular lens was implanted in all cases. Post procedure Macular thickness was measured using swept source OCT at 1^st^ postoperative day, 1st month and 6th month after surgery.

**Results:**

The mean age of patients was 62.06 ± 5.1 years. Total of 52 eyes diagnosed with senile cataract were included in this study. There were 30 (57.7%) males and 22(42.3%) females. The mean preoperative central foveal thickness was 201.3±24.8μm. The postoperative central foveal thickness was 200.3±25.2μm (153–265μm) at day 1^st^ of surgery, 224.1 ± 53.8 μm (151–458 μm) at 1st month and 212.4±28.3μm (167–255μm) 6th month after surgery. The mean preoperative BCVA was 0.70 ± 0.43 (0.1–1.7) logMAR. The postoperative mean BCVA was 0.26 ± 0.42 (0.00–3.10) logMAR at 1st day, 0.07± 0.10 (0.000.7) logMAR at 1st month and 0.05 ± 0.10 (0.00–0.3) logMAR at 6th month.

**Conclusion:**

In our study we found an increase in macular thickness but there was no loss of BCVA from changes of macular thickness after surgery and the mean BCVA increased progressively in postoperative period.

## INTRODUCTION

Cataract is one of the most common causes of decreased vision all over world, and treated only surgically.^1^ Phacoemulsification is the most preferred procedure for cataract treatment. Macular edema is one of the common and potential complications following uncomplicated cataract surgery in patients that interferes with better visual outcomes.^2^ Pseudophakic cystoid macular edema (Irvine-Gass syndrome) caused by movement and damage in the vitreous cavity and release of inflammatory mediators due to the damage of blood-aqueous barrier.^3^-^5^ There is also a role of surgical trauma in pseudophakic macular edema, by release of prostaglandins, blood retinal barriers disruption and perifoveal vascular leakage.^6^ Pseudophakic macular edema remains the main and most frequent cause of unfavorable visual outcome after uneventful cataract surgery.^7^,^8^ Optical coherence tomography (OCT) has been used to study macular changes after uncomplicated cataract surgery and the results vary in different studies, retinal thickness is increased in some studies^9^,^10^ whereas in others a decrease is reported.^11^

In light of the disparity in the data assessing the effect of phacoemulsification on macular thickness mentioned above, this study aims to evaluate the impact of uneventful phacoemulsification on macular thickness. The results of international studies may not be applicable to our population because of the different racial makeup, this study will add to the local literature and may help in improving our current understanding to the role of cataract surgery in increasing thickness of macula which interferes Post-operative vision.

## METHODS

The study was conducted at Department of Ophthalmology, Jinnah Post-Graduate Medical Centre, Karachi from 10^th^ December 2019 to 10^th^ June 2020. Sample size calculation was done by using open epi software with the following assumptions of Confidence interval (1-α) = 95%, Level of significance (α): 0.05, Power: 0.80, by taking preoperative mean central foveal retinal thickness of 202.4 ± 25.9 μm and post operatively mean central foveal retinal thickness of 226.2 ± 54.9 μm.^12^ Sample size calculated was 52 eyes. Approval of the study was done by institutional review board and ethical committee of Jinnah Post Graduate Medical Centre (Ref: No.F.2-81/2021-GENL/53744/JPMC, Dated: 03-02-2021). A pre-designed proforma was filled. Patients of either gender, age between 50 to 75 years having senile cataract with informed consent and no preexisting ocular disease were included in the study. Patients with any comorbid, ocular trauma, having pre-existing ocular diseases such as active ocular infection, glaucoma, maculopathy or retinopathy were excluded from the study. After complete history all patients underwent detailed ophthalmologic examination and Pre-surgery macular thickness recorded by using swept source OCT (DRI-OCT-2 Triton; Topcon). Phacoemulsification was performed and intraocular lens was implanted in all cases. Post procedure Macular thickness was measured using swept source OCT at 1^st^ postoperative day, 1st month and 6th month after surgery. SPSS version 21 was used for data entry and analysis. Mean (SD) was computed for Age, pre and post-surgery macular thickness. Frequencies and percentages were Calculated for categorical variables like age, gender, co-morbidity (Hypertension [blood Pressure >160/100], Diabetes [HbA1c >7], Hep B [positive HbsAg], Hep C [Positive Anti HCV]. Pre and post-surgery macular thickness were compared using paired T test. Effect modifiers like age, gender, co-morbidity were addressed through stratification. Post stratification, Dependent/paired T test was applied. P- value ≤ 0.05 was considered significant.

## RESULTS

**Fig.1:**
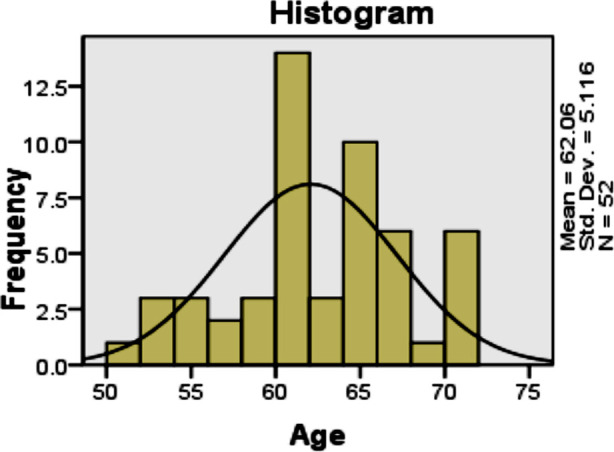
Age distribution of the patients.

A total of 52 eyes with diagnosed cataract were included in this study. Age distribution of the patients is presented in Fig.1. There were 30 (57.7%) males and 22(42.3%) females as shown in Table-I and Fig.2.

**Table-I:** Central foveal retinal thickness measurements (circle with 1-mm diameters).

	Mean Central Foveal Retinal Thickness (Range)	P Value
Preoperative	201.3±24.8μm	
1^st^ Day	200.3±25.2μm (153-265μm)	0.29
1^st^ Month	224.1 ± 53.8 μm (151–458 μm)	<0.001
6^th^ Month	212.4±28.3μm (167-255μm)	<0.001

Mean change in central foveal thickness preoperatively and after uneventful phacoemulsification using swept source optical coherence tomography is shown in Table-II. The mean preoperative central foveal thickness was 201.3±24.8μm. The postoperative central foveal thickness was 200.3±25.2μm (153–265μm) at day 1^st^ of surgery, 224.1 ± 53.8 μm (151–458 μm) at 1st month and 212.4±28.3μm (167–255μm) 6th month after surgery.

**Table-II:** Macular thickness measurements at the perifoveal four quadrants (between two circles with1mm and 3-mm diameters).

	Superior	Inferior	Nasal	Temporal
Preop.	263.8± 26.7μm	265.1± 28.4μm	261.2± 33μm	251.7± 30.2μm
1^st^ Day	262.5± 24.7μm (p=0.8)	263.6± 26.3μm (p=0.73)	261.7± 25.6μm (p=0.91)	251.3± 24.2μm (p=0.96)
1^st^ Month	285.7 ± 26.3 μm (p<0.001)	286.1 ± 24.1 μm (p<0.001)	284.6 ± 31.1 μm (p<0.001)	270.2 ± 30.3 μm (p<0.001)
6^th^ Month	280.1± 17.5μm (p<0.001)	282.1± 17.7μm (p<0.001)	278.3± 22.4μm (p<0.001)	268.7± 21.3μm (p<0.001)

Macular thickness measurements at the perifoveal four quadrants preoperatively, 1^st^ day of surgery, 1st month and 6th month after surgery are shown in Table-III. The mean perifoveal macular thickness increased in each quadrant at postoperative visits. The change in perifoveal macular thickness measurements in the temporal, nasal, superior, and inferior quadrant was statistically significant at postoperative 1st month and 6^th^ months (*p* < 0.001, for all measurements).

**Table-III:** The mean best corrected visual acuities in LogMAR.

	Mean Best Corrected Visual acuities (Range)	P Value
Preoperative	0.70 ± 0.43 (0.1–1.7)	
1^st^ Day 1^st^ Month	0.26 ± 0.42(0.003.10) 0.07 ± 0.10 (0.000.7)	<0.001 <0.001
6^th^ Month	0.05 ± 0.10 (0.000.3)	<0.001

The mean preoperative BCVA was 0.70 ± 0.43 (0.1–1.7) logMAR. The postoperative mean BCVA was 0.26 ± 0.42 (0.00–3.10) logMAR at 1st day, 0.07± 0.10 (0.0007) logMAR at 1st month and 0.05 ± 0.10 (0.00–0.3) logMAR at 6th month shown in Table-IV. The difference between the BCVA values of preoperative and postoperative control visits were statistically significant (*p* < 0.001). BCVA of the patients displayed improvement starting from postoperative first day to sixth month.

A mild statistically insignificant decrease in central, superior, and inferior quadrant thicknesses was detected in the postoperative first day measurements compared with the preoperative values but statistically there was significant increase in postoperative 6^th^ month retinal thickness measurements that becomes static and was not found to be associated with loss of BCVA. The clinical examination of the patients with slit lamp biomicroscopy by using +90 D noncontact lens revealed clinically evident macular edema in all eyes with central macular thickness approximately ≥250 μm.

## DISCUSSION

In our study, after uncomplicated phacoemulsification macular thickness increased from first week to 6th month as shown by OCT. Nasreen et al.^13^ conducted a similar study and observed that macular thickness showed an increasing trend after uncomplicated phacoemulsification starting from first week to 6th month which was also favored by a study conducted in 2021 by Kemer et al.^14^ A study conducted by Lobo et al.^15^ showed that increase in macular thickness was initially present in perifoveal region and then it leaked and accumulated in fovea. This was analyzed by retinal leakage analyzer (RLA) which demonstrated that primary site of leakages was perifoveal vascular structure. In our study we observed perifoveal and central macular thickness post operatively and an increase in both perifoveal and CMT were found postoperatively.

Retinal thickness increase on first postoperative day was shown by studies done by Nicholas et al^16^ and von Jagow et al^17^ et al but in our study we found a mild decrease in retinal thickness on OCT at first postoperative day which was advocated by another study done by Perente et al.^12^ This decrease in retinal thickness in OCT measurements was attributed to removal of the light-scattering effect of the cataract and disruption of the optical quality of the OCT imaging or it could be related to the influence of lens opacity on the preoperative OCT measurements or to an apparent thinning of the retina when the lens is replaced by an IOL. Thus, the first postoperative day measurements might reflect the actual retinal thickness.

The Factors involved in mechanism of postoperative CME development are prostaglandin production due to free-radical release following surgical trauma, prostaglandin production in anterior segment ischemia and prostaglandin production secondary to free-radical release in postoperative period with increased light exposure to retina. Vitreoretinal adhesion causes mechanical traction also contributes in postoperative CME development. Prostaglandin and some other inflammatory factors are accumulating in the aqueous humor, penetrate the vitreous body and altered blood retinal barrier permeability at the macula, with an accumulation of fluid in extracellular spaces. The development of CME in eyes without mechanical traction and even without posterior vitreous detachment increased the importance of prostaglandin theories. Therefore, prevention and management of CME by inhibition of prostaglandin production were tried.^18^-^21^ These theories have important role in the prevention and treatment of long term macular changes. There are several other factors which could affect postoperative macular state, such as diabetes, smoking, type of the implanted IOL, adrenalin injection into the anterior chamber, surgical technique and duration of procedure.

The incidence of cystoid macular edema is estimated 0.1% to 7.0%^6^,^7^,^21^ and is known to be the most important reason behind postoperative decline in visual acuity after cataract surgery. Our study shows no effect on BCVA from changes of macular thickness and the mean BCVA increased progressively in postoperative period.

### Limitations:

Although our study has shown promising result but it is limited in terms of number of patients, duration and number of follow ups to assess long term effects. Study has shown variability of the operating surgeon.

## CONCLUSION

In conclusion, using high-tech diagnostic devices such as OCT, it is possible to detect subclinical macular edema after uncomplicated phacoemulsification. In our study we found an increase in macular thickness but it was not large enough to reduce visual acuity so no loss of BCVA from changes of macular thickness after surgery and the mean BCVA increased progressively in postoperative period. The consequences of chronic CME should be avoided. Medical treatment of CME should be done at early stages before secondary problems such as clinically significant macular edema, macular hole, or epiretinal membrane formation occur. There should be comparative studies with proper follow up to conclude the most effective management plan.

### Authors’ Contributions:

**MD:** Conceived the study and managed data collection.

**MAT:** Study was done under his supervision, edited, corrected and finalized manuscript. And he is responsible for the accuracy or integrity of study.

**AC:** Contributed in acquisition of data, critical review and approval of manuscript.

**HNN:** Contributed in study design and drafting the article, contributed in data acquisition.
